# Postpartum intentions on contraception use and method choice among breastfeeding women attending a university hospital in Ohio: a cross-sectional study

**DOI:** 10.1186/s12978-017-0307-4

**Published:** 2017-03-20

**Authors:** Yiska Loewenberg Weisband, Lisa M. Keder, Sarah A. Keim, Maria F. Gallo

**Affiliations:** 1 The Ohio State University, College of Public Health, 1841 Neil Ave, Columbus, OH 43210-1351 USA; 2 The Department of Obstetrics and Gynecology, Ohio State University, Columbus, OH 43210 USA; 3 Center for Biobehavioral Health, The Research Institute at Nationwide Children’s Hospital, Nationwide Children’s Hospital, 700 Children’s Drive, Columbus, OH 43205 USA

**Keywords:** Breastfeeding, Contraception, Lactation, Postpartum, Unintended pregnancy

## Abstract

**Background:**

Few postpartum women use effective contraception and those who use less effective methods have increased rates of unintended pregnancy. Little is known about postpartum contraception intentions among breastfeeding women. Our objectives were to measure the extent of prenatal contraceptive counseling, to assess contraceptive intentions, and to identify correlates of both among postpartum women who were planning to breastfeed.

**Methods:**

We conducted a cross-sectional study using a convenience sample of 100 breastfeeding women before their discharge following delivery at a large university hospital in 2015. We used logistic regression to assess three outcomes of interest: not intending to use contraception before 6 months postpartum, reporting receiving counseling on postpartum contraception during prenatal care, and considering the effects of contraception methods on the breastfeeding mother-infant dyad when choosing a postpartum contraception method.

**Results:**

Most women (91%) intended to use contraception. Prior history of no contraception use was the sole factor related to not intending to use contraception. The most commonly cited reason for the intended choice of contraceptive method was convenience (35%). Few women (21%) reported considering the effects of contraception methods on the breastfeeding dyad when choosing a postpartum contraception method. Nearly half of women reported never discussing postpartum contraception options with their healthcare provider during prenatal care. In the multivariate analysis, receiving public assistance was the only factor that remained statistically significantly associated with reporting having received contraception counseling during prenatal care.

**Conclusions:**

Although most women intended to use contraception, they did not appear to have received adequate prenatal counseling on postpartum contraception.

## Plain english summary

Few women after pregnancy use effective contraception. Women who do not use an effective form of contraception experience higher rates of unplanned pregnancy. Little is known about the intentions to use contraception among breastfeeding women after delivery. Our objectives were to measure how often women remembered being counseled on contraceptive methods during prenatal visits, to assess contraceptive intentions, and to identify factors that were associated with these issues. We conducted a cross-sectional study using a convenience sample of 100 breastfeeding women before their discharge following delivery at a large university hospital in 2015.

Most women planned to use contraception. Women who had not used contraception in the past were less likely to report that they planned to use contraception. The most commonly cited reason for choosing a contraceptive method was convenience. Few women reported that the effect a method may have on her or her infant influenced her choice of method. Nearly half of women reported never discussing their options for contraception methods with their healthcare provider during prenatal care. Women who received public assistance were 5 times more likely to report having received prenatal counseling regarding postpartum contraception than women who did not receive public assistance. Despite the fact that most women were interested in using a form of contraception after pregnancy, most women did not recall receiving counseling on this issue. Our results highlight the need for high quality prenatal counseling for all women, to reduce the likelihood of unintended pregnancies and poor spacing between pregnancies.

## Background

Effective contraception use among postpartum women is critical for preventing adverse perinatal outcomes resulting from inadequate inter-pregnancy intervals [1–4]. Unmet need for postpartum contraception, though, remains high in many settings: an analysis of Demographic and Health Surveys conducted in 21 low- and middle-income countries estimated that 61% of postpartum women did not want to become pregnant in the next year and yet were not using contraception [5]. Data from the U.S. National Survey of Family Growth show that few postpartum women use long-acting contraception and that those who use less effective methods have increased rates of unintended pregnancy [6]. Interactions with the healthcare system around the time of childbirth could represent an important window for intervening as women might be highly motivated to initiate postpartum contraception use to prevent a closely-spaced, repeat birth. Initiating long-acting reversible contraception (LARC) during the early postpartum period could effectively delay repeat pregnancies [7]. Furthermore, bundling contraception counseling and provision into pregnancy visits can be cost-effective and efficient [8]. Accordingly, the World Health Organization (WHO) recommends postpartum women be offered contraceptive counseling and provision following delivery before discharge [9], and professional associations in the U.S. identify prenatal visits as an ideal window for providing counseling on postpartum contraception [10].

In the case of breastfeeding women, these services should include counseling on modern methods that are safe to use while nursing, as not all methods are recommended in the early post-partum period [11, 12]. Guidelines from the Center for Disease Control (CDC) indicate that breastfeeding women who are less than 6 weeks postpartum should not use combined oral contraceptives, but that the advantages of the use of progestin only pills, implants and injectables outweigh any theoretical or proven risks associated with these methods [11]. In contrast, WHO does not recommend use of injectable contraceptives among breastfeeding women prior to 6 weeks postpartum [12].

Among the general population, the factors influencing women’s intentions to use contraception have been studied extensively [13–16]. However, less is known about the determinants of contraception intentions among postpartum women, in general, and among breastfeeding women, specifically [17–19]. Our objective was to measure – before their hospital discharge – any contraceptive intentions and the extent of prenatal contraceptive counseling among postpartum women who were planning to breastfeed and to identify correlates of both.

## Methods

### Study setting and design

We conducted a cross-sectional study using a convenience sample of 100 postpartum women at a large university hospital in central Ohio during September to November 2015. The study was part of a preliminary study to assess feasibility for a future randomized control trial. To be eligible for participation, women had to meet the following inclusion criteria: be ≥18 years of age, speak English, have delivered a term, healthy, singleton infant of ≥2500 g, plan to nurse for ≥6 months, and still be a patient in the postpartum ward. No exclusion criteria were applied. The study consisted of a single questionnaire administered by a trained interviewer. All women were interviewed within 72 h of delivery, prior to being discharged from the hospital. In order to minimize the inconvenience to the postpartum women, the interviewer read the questions aloud to the participant and recorded her responses directly into a laptop computer using REDCap software, a secure, web-based application for electronic data capture. All participants provided written consent before participation. The Ohio State University institutional review board approved the study.

### Study instrument

The questionnaire covered the following domains: socio-demographic characteristics, contraceptive history (method, type and preferences), intention to start a method in the next 12 months (any intention, method intended to use, intended timing of initiation, and rationale for selected method), knowledge of contraindications to postpartum contraception use during breastfeeding, and recall of counseling during prenatal care on postpartum contraception. For the questions regarding contraceptive methods, participants were read a list of contraceptive methods and could choose all that applied.

### Outcomes of interest

We assessed three outcomes of interest: 1. No intention to use contraception before 6 months postpartum, 2. Reporting receiving counseling on postpartum contraception during prenatal care, and 3. Considering the effects of contraception methods on the mother-infant breastfeeding dyad when choosing a postpartum contraception method.

Having no intention to use contraception before 6 months postpartum was assessed using the following questions: “Do you intend to use contraception” and “when do you intend to begin?” Women were categorized as not intending to use contraception before 6 months postpartum if they responded that they did not intend to use contraception, or if they responded that they intend to use contraception, but intend to begin the use of a method after 6 months.

Reporting receiving counseling on postpartum contraception during prenatal care was assessed using the following question: “Did any of your health care providers discuss your postpartum options for contraception with you?” Participants who answered “yes, during prenatal care” were categorized as having received counseling on postpartum contraception during prenatal care.

Considering the effects of contraception methods on the breastfeeding dyad when choosing a postpartum contraception method was assessed using the following question: “Why did you choose this method?” Women who who gave an answer consistent with “safe for the breastfeeding mother” or “safe for breastfeeding infant” were categorized as considering the effect of the method on the breastfeeding dyad.

### Potential correlates

The following were evaluated as potential correlates for the 3 outcomes: age (continuous variable), race (non-Hispanic white vs. other), relationship status (married vs. unmarried), education (no college vs. at least some college), employment in past year, receive public assistance in past year – unemployment benefits, food stamps and/or free lunches at school, having Medicaid coverage, having a prior birth, and ever using contraception.

### Data analysis

We report descriptive findings using proportions and means with standard deviations. We used logistic regression to identify correlates of 3 outcomes of interest: not intending to use contraception before 6 months postpartum, reporting receiving counseling on postpartum contraception during prenatal care, and considering the effects of contraception methods on the breastfeeding dyad when choosing a postpartum contraception method. We fit bivariable logistic regression models for each potential correlate with each of the three outcomes of interest (in separate analyses). We then fit a full model with the correlates that were statistically significantly related in the bivariable analysis with the given outcome of interest based on a p-value of <0.10 and, in a backward stepwise progression, manually removed variables that were not associated in the adjusted analysis. Statistical significance was based on a p-value of <0.05. Analyses were performed using STATA software (release 12; Stata, StataCorp, College Station, TX, USA).

## Results

We recruited 102 eligible women, of whom two declined to participate. Thus, we enrolled 100 (98% of eligible) women. Participants had a mean age of 30.4 ± 5.0 years. Most were non-Hispanic white (76%), married (73%), had at least some college education (82%), and had ≥1 prior live birth (58%) (Table 1). Overall, 27% of the participants were enrolled in Medicaid. Prior contraception use was common (93%) with condoms (28%) or combination oral contraception (24%) as the most common method at last use (Table 1).

**Table 1:** Demographic, socioeconomic and reproductive characteristics among postpartum women planning to breastfeed for ≥6 months, *N =* 100

	%
Age	
18–24	12
25–34	69
35+	19
Race/ethnicity	
Non-Hispanic white	76
Non-Hispanic black	10
Hispanic	5
Other	9
Relationship status	
Married	73
Cohabiting	13
Not married or cohabiting	14
Highest educational level attained	
High school or technical school	18
Some college or college degree	55
Some graduate school or graduate degree	27
Employment in past year	
Full time	65
Part time	22
Not employed	13
Received public assistance in past year	
Yes	19
No	81
Medicaid coverage	
Yes	27
No	73
Number of prior live births	
0	42
1	38
2 or more	20
Last contraceptive method used^a^	
Condom	28
Combination oral contraceptive	24
Withdrawal	14
Intrauterine device	11
Other methods^b^	10
Rhythm	7
Progestin-only pills	6
No history of contraception use	7

^a^Participants could give >1 response; thus, sum exceeds 100%

^b^Consisted of injectable (4%), contraceptive ring (2%), implant (1%), contraceptive patch (1%), diaphragm (1%) and spermicide (1%)

Most respondents (91%) reported intention to use contraception postpartum (Table 2). Among those reporting this intention, 55% planned to begin using a method before hospital discharge or within 6 weeks postpartum. An additional 35% intended to begin the use of contraception between 6 weeks and 6 months. Intrauterine devices (IUDs) were the most commonly reported planned contraceptive method (24%), followed by condoms (23%), combination oral contraception (13%), and progestin-only contraception (12%). Among women who reported planning to begin using a method before hospital discharge, the most common method choices were female sterilization (38%) and implants (38%), followed by injectables (12%), male sterilization (6%) and intrauterine devices (IUD) (6%). Only 21% of women indicated breastfeeding safety as the reason for choosing the type of method they wanted to use. Among women who stated non-breastfeeding-related reasons for choosing the type of method they intended to use, the most common reason cited was convenience (35%).

**Table 2:** Contraception intentions among postpartum women planning to breastfeed for ≥6 months, *N =* 100

Intention	No.	(%)
Intend to use contraception		
Yes	91	(91)
No	9	(9)
Intended contraceptive method^a,b^		
Intrauterine device	22	(24)
Condom	21	(23)
Combination oral contraceptive	12	(13)
Progestin-only pills	11	(12)
Female sterilization	10	(11)
Male sterilization	9	(10)
Implant	9	(10)
Injectable	7	(8)
Withdrawal	3	(3)
Lactational amenorrhea	2	(2)
Fertility awareness-based method	2	(2)
Contraceptive patch	1	(1)
Unsure	8	(9)
Planned timing of contraception initiation^a^		
Before hospital discharge	16	(18)
In the next 6 weeks	34	(37)
Between 6 weeks and 6 months	35	(39)
Between 6 months and 1 year	3	(3)
Over a year or unsure	3	(3)
Intended method choice was related to safety during breastfeeding^a,c^		
Yes	18	(21)
No	68	(79)

^a^Among those intending to use contraception (*n =* 91)

^b^Participants could give >1 response; thus, sum exceeds 100%

^c^Missing 5 responses (*n =* 86)

Only 57% of the women reported that their healthcare provider discussed postpartum contraception options with them during prenatal care (Table 3). Most women could not correctly identify the contraceptive methods that are recommended for use according to the CDC, among breastfeeding women during the first 6 weeks postpartum [11, 12]. Only 43% correctly identified combination oral contraceptives as contraindicated for lactating women during this interval. Similarly, a minority of women correctly identified IUDs (42%), progestin-only pills (42%), implants (35%), and injectables (28%) as being recommended for use during this period. Most women (82%) could correctly identify at least one method that was recommended for use during the first 6 weeks postpartum.

**Table 3:** Contraception counseling and knowledge about recommended postpartum contraceptive methods among postpartum women planning to breastfeed for ≥6 months, *N =* 100

Characteristic	%
Reporting receiving counseling during prenatal care on postpartum contraception	
Yes	57
No	43
Correctly identified methods not recommended for postpartum use during first six weeks of breastfeeding	
Combination oral contraception	43
Correctly identified methods recommended for postpartum use during first six weeks of breastfeeding	
Progestin-only pills	42
Intrauterine device	42
Implant	35
Injectable	28

Age and lack of history of contraception use were the sole factors related to not intending to use contraception before 6 months postpartum; women who had no prior history of contraception use had 5.1 times the odds of not intending to use contraception by 6 months postpartum, compared to women who had used contraception in the past (OR 5.1; 95% CI: 1.0, 25.5), and the odds of not intending to use contraception before 6 months postpartum increased as women’s age increased (OR 1.2; 95% CI: 1.0, 1.3). Only age remained significantly associated with not intending to use contraception before 6 months postpartum (AOR 1.2; 95% CI: 1.0, 1.3) (Table 4). We found three statistically significant factors associated in the bivariable analysis with higher odds of reporting receiving counseling during prenatal care on postpartum contraception use: 1) Hispanic Women and non-White women had 2.9 times the odds of reporting receiving contraceptive counseling compared to non-Hispanic White women (OR 2.8; 95% CI: 1.0, 7.9); 2) Women who received public assistance had 5.2 times the odds of reporting receiving contraceptive counseling compared to women who did not receive public assistance (OR 5.2; 95% CI: 1.4, 19.2); and 3) Women who had Medicaid coverage had 3.6 times the odds of reporting receiving contraceptive counseling compared to women who did not have Medicaid coverage (OR 3.6; 95% CI: 1.3, 9.9) (Table 4). In the multivariable analysis, receiving public assistance was the only factor that remained statistically significantly associated with reporting this counseling.

**Table 4:** Correlates of no intention to use contraception, receiving contraception counseling and contraceptive method related to breastfeeding safety, from bivariable logistic regression

	No intention to use contraception before 6 months postpartum		Received counseling during prenatal care on postpartum contraception		Contraceptive method choice related to breastfeeding safety	
Potential correlate	OR	(95% CI)	OR	(95% CI)	OR	(95% CI)
Age	**1.2**	(**1.0**, **1.3)**	1.0	(0.9, 1.1)	1.0	(0.9, 1.1)
Race/ethnicity						
Non-White	1.8	(0.5, 5.7)	**2.8**	**(1.0, 7.9)**	0.3	(0.1, 1.6)
White	1		1		1	
Relationship status						
Married	6.2	(0.8, 49.4)	0.6	(0.2, 1.4)	1.0	(0.3, 3.0)
Unmarried	1		1		1	
Highest educational level attained						
No college	- ^a^		1.6	(0.6, 4.8)	1.4	(0.4, 4.9)
Some college or college degree	1		1		1	
Employment in past year						
Full time	2.4	(0.6, 9.2)	1.0	(0.4, 2.3)	1.1	(0.4, 3.2)
Not employed or part time	1		1		1	
Public assistance in past year						
Yes	0.6	(0.1, 3.0)	**5.2**	**(1.4, 19.2)**	0.5	(0.1, 2.3)
No	1		1		1	
Medicaid coverage						
Yes	0.6	(0.2, 2.5)	**3.6**	**(1.3, 9.9)**	**0.1**	**(0.0, 1.0)**
No	1		1		1	
Primiparous						
Yes	1.7	(0.6, 5.2)	0.52	(0.2, 1.2)	1.1	(0.4, 3.2)
No	1		1		1	
Ever contraception use						
Yes	1		1		1	
No	**5.1**	**(1.0, 25.5)**	4.9	(0.6, 42.7)	0.7	(0.1, 6.6)

All correlates associated at the 0.1 level were included in a full multivariable model; Bold values indicate variables that remained significant in the multivariable analysis, after backward stepwise regression

*CI* confidence interval, *OR* odds ratio

^a^ – observations dropped since they perfectly predict failure

Having Medicaid coverage was the only factor that significantly reduced the odds of considering the effects of contraception methods on the breastfeeding dyad when choosing a postpartum contraception method (OR 0.1; 95% CI: 0.0, 1.0).

## Discussion

In this study of postpartum, breastfeeding women surveyed before hospital discharge, intention to use contraception was high. The most commonly cited reason for their intended choice of contraceptive method was convenience. Surprisingly, few women reported considering the effects of contraception methods on the breastfeeding dyad, either in terms of the effects on the mother or the infant, as a motivator in their choice of postpartum contraceptive method. Women had limited knowledge regarding the methods that are recommended for use during the first six weeks postpartum while breastfeeding, and nearly half of women reported never discussing postpartum contraception options with their healthcare provider during prenatal care. In the adjusted analyses, women who reported having received contraception counseling from their healthcare provider during prenatal care visits were more likely to receive public assistance.

Fifteen percent of participants in our study reported having no intention to use contraception before six months postpartum. Given the substantial decline in efficacy of lactational amenorrhea by six months postpartum, women who do not initiate contraception during this interval are at an increased risk of having an unintended pregnancy [20]. Furthermore, many women who plan to breastfeed do not follow through with their plans: one US study found that 22% of women who were planning on breastfeeding their newborns at the time of discharge from the hospital discontinued before six weeks postpartum [21]. Discontinuation of breastfeeding could leave women who were planning to rely on protection from lactational amenorrhea at risk for an unintended, repeat pregnancy. Ensuring adequate inter-pregnancy spacing is especially important because of the association between short intervals and increased risk of having a small-for-gestational-age infant, pre-term birth, and neonatal morbidity [2–4]. Accordingly, the WHO recommends that women delay attempts to conceive a repeat birth for ≥24 months following a live birth [22].

Although over 90% of women in the present study reported an intention to use contraception, nearly half reported never having discussed postpartum contraception options with their healthcare provider during prenatal visits. This lack of counseling could lead to a gap in knowledge regarding the safety and efficacy of different methods during the postpartum period. Women receiving public assistance were more likely to report the receipt of contraceptive counseling during prenatal care than other women. Health care providers may view these women as being at a higher risk for an unintended pregnancy and therefore target them for this counseling. Previous studies of postpartum women, found that less than 25% of women reported a lack of prenatal contraception counseling [19, 23]. One of these studies, which also interviewed women before discharge from an urban university hospital, found that black race, lack of college education and receiving prenatal care at a resident clinic were positively associated with reporting having received prenatal contraception counseling. The reasons for the lower frequency of reporting receipt of this prenatal counseling in the present study are unknown. A lack of counseling could leave women ill-informed about the timing of the return of fertility and insufficiently motivated to initiate postpartum contraception use.

The American College of Obstetricians and Gynecologists and the American Academy of Pediatrics advise that discussion of contraceptive options and prompt postpartum contraception initiation be included in prenatal and postpartum care [10]. Both professional associations further recommend that contraceptive counseling ideally should occur during prenatal visits, as women are typically more focused on other concerns at the postpartum visit. A recent meta-analysis found inconsistent evidence on whether providing postpartum contraceptive counseling reduces unmet need or repeat pregnancy rates [24]. However, the authors assessed the quality of evidence on intervention effectiveness as being mostly low, and recommend more rigorous trials be conducted to determine the effects of the timing and elements of postpartum contraception counseling on contraception use and unintended pregnancy rates.

Inadequate knowledge on contraception is associated with incorrect or inconsistent use of contraception and method discontinuation [25]. Our findings indicate very limited knowledge regarding which methods are recommended for use during the first six weeks of lactation. This may lead to women choosing a method that could undermine their breastfeeding goals. Improved matching of women to contraceptive methods based on breastfeeding goals might help meet national breastfeeding goals for duration and exclusivity.

Despite the limited knowledge the women in our study displayed regarding recommended contraceptive methods during the first 6 weeks postpartum, they were more likely to correctly identify whether their intended contraceptive method was recommended for use among postpartum breastfeeding women (data not shown). This is consistent with previous studies, which have found that women had greater knowledge about the method that they had chosen to use [13].

Few studies have assessed reasons why women choose their intended postpartum method of contraception. A study of pregnant and postpartum women found that many changed their method postpartum as compared to what they used before pregnancy, with the most predominant reason cited being to avoid repeat births followed by safety concerns for mother or baby, method reliability, and an increased desire for a long-term contraception [17]. Previous studies, to our knowledge, have not focused specifically on postpartum women who intend to breastfeed. Surprisingly, women in the present study did not commonly report concerns about breastfeeding compatibility of the contraceptive method as a factor in their method choice. Women who had Medicaid coverage were less likely to state breastfeeding compatibility as a reason for choosing their contraceptive method. In general, participants reported effectiveness and convenience as attributes influencing their method choice.

The primary limitation to the interpretation of these findings is the sample size; low study power could have prevented the identification of important correlates of the outcomes of interest. In addition, because study participants were relatively highly educated and were recruited from a single urban hospital, findings may not be generalizable. Finally, the use of in person interviews may have led to interviewer bias, however, the interviewer was trained and followed a strict protocol to reduce the likelihood of such bias. The strength of the study was its focus on postpartum women intending to breastfeed before their hospital discharge; the timing of the interview offers a unique assessment of women’s intentions immediately postpartum.

Women in the present study might have failed to report the receipt of postpartum contraceptive counseling during prenatal care – despite its occurrence – because of poor recall. This suggests the need for improved quality of counseling to increase its retention and role in women’s decision-making. Future research should determine methods for ensuring the delivery of high-quality, effective contraceptive counseling and identify the ideal times for providing this counseling. Research also is needed to harmonize the CDC and WHO recommendations concerning the use of injectable contraception immediately postpartum among breastfeeding women to ensure that postpartum women have the widest range of possible options to prevent unintended pregnancy and ensure adequate birth intervals.

## Conclusions

Despite clear guidelines recommending the discussion of contraceptive options in prenatal care, and the overwhelming majority of women who intend to use contraception, few women in our study reported receiving counseling regarding postpartum contraception options during their prenatal care. All women could benefit from counseling, which could increase the use of effective methods recommended for breastfeeding women, thereby decreasing the likelihood of unintended pregnancy and inadequate inter-pregnancy intervals. Improving the implementation of these guidelines could reduce adverse perinatal outcomes resulting from inadequate inter-pregnancy intervals.

## Abbreviations

CDC: Center for disease control
IUD: Intrauterine devices
LARC: Long-acting reversible contraception
WHO: World Health Organization
